# Supplementary material for: Patterns of Intron Gain and Loss in Fungi
Source: PLoS Biol. 2004 Nov 30;2(12):e422. doi: 10.1371/journal.pbio.0020422 (PMC532390; doi:10.1371/journal.pbio.0020422)
Supplement: Table S1 — Also available at http://genes.mit.edu/NielsenEtAl/. (4.3 MB ZIP). [file pbio.0020422.st001.zip › NielsenEtAl/html/501.html]

AN0407.1.NCU00310.1.MG06314.1.FG05376.1


```
 CLUSTAL W (1.82) Multiple Sequence Alignments - Introns Inserted


Sequence 1: NCU00310.1	1142 aa
Sequence 2: MG06314.1	1071 aa
Sequence 3: FG05376.1	934 aa
Sequence 4: AN0407.1	1077 aa
Alignment Length: 1211 aa
Number Identitical Residues: 158 aa
Alignment Score (without introns) 10388


MG06314.1 	-------MDNPYP~EEALLDNHQLIH----------QDEQLGQTVFTREDFEYALRD2FQ
NCU00310.1	MDDDIMDIDDNSP~AHYAGDGQSGVHSAAYFYNQYDDDIPLDPELMAGDDDQMDGLD~DY
FG05376.1 	-------MDMMER2QHEN---------------------------------------~--
AN0407.1  	----MDYFGEDDP~NHSPHLGEIAYPDIDETVQYPWQGSGQSFSHEASAIDPRLYKD~LF
          	       :.      .    .     .     .   ..   .    :         .   

MG06314.1 	EMSGG--EDGGAVSEVSDADSVYLGVQEQINRAQADRTAFLDAFQN~PQEDDDEDIYDGL
NCU00310.1	DLDDEQMQDGFVDPEEEFIDSDTEEIRAEIHKFETVRDAWLAEHRA~DLPSAAYTVLPGV
FG05376.1 	---------------ASDADSDLEELEDDIAKFDQTVRNFINEQR-~-----DEVVPPSA
AN0407.1  	SGGESQEPTQSAADEEQFAPEEEQSLELDNSDESSSFVASSEEETS2EDLSDDDAPGSDQ
          	. .  .        . .   .    :. :    .                .       . 

MG06314.1 	PTKPPPRKK--VKRG---------PVVRKG-PPIKKAPRKAKELPTEIKVLVDAATNAFI
NCU00310.1	SKSMSKQINNSLKDGSTTTVAPSAPAPRPSRPRRKLGPRKPPRPPPEIQIRIAAARTAFD
FG05376.1 	NRSGLRGKG-------------------------ARGPRKAAKPRGDITARLSKVNQAFL
AN0407.1  	EDGLRRRRRRRGRFSG--------RFGARGGKGIKRGPRKPVEPSPEFKLLHSEATSAFI
          	              ..             .      .***. .   ::      .  ** 

MG06314.1 	ANRYDEALQAARETIRLNAEVAPAWGILAAVYEEREDWRHALEAKRIQASLTKGDTQLWL
NCU00310.1	RREYQEAINMVKEIIRQRPATPQAWNLLSLIHEELGNREAATMCLISGAWLIPKDARHWM
FG05376.1 	SGDYSLALDLVFEVIRINAETHQAWTTLASIFGEQGDMSKSLSAMVYAAHLRPKDVNGWL
AN0407.1  	DGDYDRAIDLVRRAIQVNPEMFAAHSLLSEIFLAQGEKEKAVTALFSGAHTRPRDTTVWL
          	   *. *:: . . *: ..    *   *: :.    :   :  .    *     *.  *:

MG06314.1 	GTADLALHMVDGAYQDSPEEIDKTLKVAMDCYRSVLQIDKTNPVARLGKADILADLGQSS
NCU00310.1	NVALYCLYGVD-MMDDTDPRRKLALERAVMCYSQALQADKQNVEARTGKADALMQLGQSH
FG05376.1 	RCASFAL-------ENTSDDEAGNLNTARLCYSAALRADHTNLDARLGKGLVCHRQGHLA
AN0407.1  	KVARMIT-------EQAGDDRQAVLNDVLYCYSRVLEIDPQNHNTRFQRAALYRGLGHNG
          	  *           :::       *: .  **  .*. *  *  :*  :.      *:  

MG06314.1 	KAVAAYLDYLKQKPYNLRVVRSLAEHAYNARRAKEAIEATVLAYEACIQHFKS-GQTLDD
NCU00310.1	LALAQYLRALKVQPLNIRTVRNLAEAALDGRDPRKNAEAAKAAYRHIIEYLWSQGDEVYE
FG05376.1 	AAISDYKVVLKHRPYDLEIVRKLAEACVDNKQAEAAVPSAIAAYKRFFDHEKK--ETSLE
AN0407.1  	RAVTEYERILRDLPHNVRALRLLTDTLSEQNQYQKALDYWSESIQHYMAQEPE-------
          	 *:: *   *:  * ::. :* *::   : .  .        : .  :    .       

MG06314.1 	--DQVLDWIDVRIFIELLASLEKYDEAAKWLKTLVRWLVGRNDDLLWDQCQ-DDREWDLD
NCU00310.1	AMEGEFEWSDLRIYLEFFTILEQWEQGAAELKRVARWKLGRRAEEFWDRWPGEDREWDED
FG05376.1 	--MIESPWTDIGIYVELFASTGRYQDAIQEAKALARWVLGRESEGYWNNWHSDDREWDMD
AN0407.1  	-ETPEFTWSDANIYIELYTYLGRHAEGLKAAKAVSRWLLGRKDDTMWDDFDEDDREWDHA
          	       * *  *::*: :   :  :.    * : ** :**. :  *:    :*****  

MG06314.1 	DT-RRLEMEGFNQGEFLPQSYGHGLPLELRGKLAVYRFRTDM--EDEAM0RHLRQLDPED
NCU00310.1	DE-RRELCEDYEPGRFSPVQYGRALPVDLRARLYVFRMKMGD--IYEAE~KHLTSLDPSA
FG05376.1 	DE-RRTGVPEFRAGLWSTDLYGRSFPLDLRARLAIYRFRLGD--EEEAM0KHLWWFDPES
AN0407.1  	DFPRRIKADGYIPKQWPPDSYGPGLPLEFRIKLGLFRLKLPERHINEAL0HHFRWLKPED
          	* .**     :    : .  ** .:*:::* :* ::*::       **  :*:  :.*. 

MG06314.1 	ADIRGKLQFTPEIAKEVADQLCESGKPERAILYYDLYRDLVGEA----LDAEYYVKRAKC
NCU00310.1	DTAVEDFYDFPDCLKDIGNALLDNESPAKALQFFSLYEKIANQAGDISMDADILVSLGRC
FG05376.1 	VITRDFANEFPFLTFDLAEELAHRGHTPLAISYYHVLRDLPGDA-----DATILLQLGRC
AN0407.1  	TSDGSLVYDYGDLFREAADALKDAEMFEDALRFYRPIQKTEYAD------VSFFMAMGDC
          	               : .: * .      *: ::   ..           .   :  . *

MG06314.1 	HVQLADGPAAEDCFIYALEVDEDNIEARYELAKMYEEAQE---RDEAFRLVGEALSLEAG
NCU00310.1	HMALGDKSAAEERFIAAIEDDEDNIEARVCLANMYEHVSDREGREEAFLLVRDAMNLEAN
FG05376.1 	HSAIGENATAEEYFLAAIDADEDNIDARIELANMYEKARE---EEEALILAAEAMALREI
AN0407.1  	FKSLGELEDAENCYLTVAEHDTSNIESRVQLAKLYESIGMT---EQALRYVNDAVLLGRQ
          	.  :.:   **: :: . : * .**::*  **::**        ::*:  . :*: *   

MG06314.1 	QSVYDG---LTHRYVVDRGKIVSKPKKYRTGATKRAPTTK----AKYRPRRLIGGGSGES
NCU00310.1	QYTLDQEGNLVPKRKRRRATGPRKPRKPRDPNRPRDPNNP----KKYVPRRLIN----AE
FG05376.1 	RDRDHNNESGQDSSTATEQPSRRAPRRTQAVPRRTDAFGKRLITRRYRPKRLAG----AE
AN0407.1  	EHRSNR--------RRKDTRLEELAREFKSVNVAAGPKDQ-----SVESSSALG------
          	.   .                   .:. :       .           .    .      

MG06314.1 	ARKKFEDEVTARLRERYTECQRLKAQMDANVAASGGHDTNDPVVAEWMSAAKELVDDFRS
NCU00310.1	KRKQVDLERTKVATKNYEIVQELQER--------AFHSDDSEAIQNWTRAARDLVEDFRS
FG05376.1 	KRRQEEQARAVKLAEQYEIVQDLRQK---------IKGGCQDLVPEWMASSKELVDDFRS
AN0407.1  	--NKGEAERTENIQFLFKKLTQLEPK---------LQAGDSEATEDWLDIADALLREFRS
          	  .: :   :      :     *. :          :   .    :*   :  *: :***

MG06314.1 	FREFYSWERYVSSLGYGNFFKAEASADGNTGDERSRSIAKMAKRLHNT1LAP~VETEGE-
NCU00310.1	FKEFYPWDKYVKYLGLG-----GTGGGGAAVPSRNLKLAAMAERLRQG~LNP~ENGDGT-
FG05376.1 	IKKFYTWDKYLHFLGPK-----NHTQQQEAGQPQN-ELSQMYERLART1LAP~QPDNGNG
AN0407.1  	NRIFYPLQRNVVFLGYS---------SKAGKDALMDELQEMANRLQES~LGA0STIPTD-
          	 : **. :: :  **                     .:  * :** .  * .        

MG06314.1 	EAVPQKIER------QEHRGIPFNDWLDLFLEYAISLARQQRIGEAYAVCQSARDSIVYG
NCU00310.1	DNIPAASKARLLKFPSAHRGIPFSAWLDIFLSLAFALVRQNQHREAYVVCHAARDSVVW-
FG05376.1 	EAGSSDLES--------HRGISFNNWLDLFLDYAIGLAIAHRRDEAYQVCEAARDSTAF-
AN0407.1  	-----------------YHGISFDEWLDLFLQYALTVAGQGEFSEAYDTLGAAADASVWY
          	                 ::**.*. ***:**. *: :.   .  *** .  :* *: .: 

MG06314.1 	KAKDSLMLIHLAWAS~CAVQAGDEETCVAIARYFMIHNPFVTDGYKMFAALTRVCQTS-P
NCU00310.1	TATDSTYLIHVAWAS~CAVYAGDEETCVAIARYFMRDYLPGTDSYRMFAAMCRTCQTP-V
FG05376.1 	QAPEHGFIIHIAWSV1CAIYTSDEEKCIATARQLMRDG-ATSDSYRMFALVSNLCQSP-V
AN0407.1  	HSRDSSRLIHVCWFT1CALRLQDEETLANEARWFIKEYQFVTDTYRLFAMLSCLSGDPHR
          	 : :   :**:.*   **:   ***.    ** :: .    :* *::** :   .  .  

MG06314.1 	TWYQSGPSQKFMLRQIRQMDEILAAKAKEEEDGFGAASNQGS------QPPGPS----AE
NCU00310.1	SWYTSGPAQKFILRQIKTMDALVMRQNGGQDEFPPPAASQRSPSKSQQQSPLKSRSRGGD
FG05376.1 	SWYTSGPAQKYILRQIKAMDEAYGESEPNEDSAKKREPS---------------------
AN0407.1  	SLFHSSPLMKFMLRQIKAIDFTLPDHIAGARHTKAIRESIYKERAALSTKDEKG------
          	: : *.*  *::****: :*                  .  .   : .     .      

MG06314.1 	TQMVPATAAAAQ-------------------PGPTG---VDAVLLVLYGCILFASTSYQY
NCU00310.1	LVAAPLTTASQQQSESNNNDVITLDNPQQHPPPPAGDITLDVALLTIYGHILFTTTSYSY
FG05376.1 	---------------------------------------LDACVLMLYGHILFTSTSYTY
AN0407.1  	--------------------------------EPIPAEEMDVALLVLYGHILYSGNSFYP
          	                                 .     :*. :* :** **:: .*:  

MG06314.1 	ALA~YFLRARTVDPDNPAINLVIGLAYIHWALKRQSHNRQYTLMQGFSYIFKYYEDRTRG
NCU00310.1	ALS~YFARAASLDPENPLINLSIGLAYVHYALKRQATNRQYLLTQGFAFLFRYYRNRTEE
FG05376.1 	ALG1YFLRSRALDPDNPMVNLSLGLAYVHYGLKRQSTNRQYLLLQGQSFLSRYAELGSSS
AN0407.1  	ALN~YFFRAYALDDKNPAVLLSIGLCYIHHSMKRQSENRHFQIMQGLSFMNEYKRLRERK
          	**  ** *: ::* .** : * :**.*:* .:***: **:: : ** ::: .* .     

MG06314.1 	--AAAPEERQEAHYNVARTYHLLGIHDLALEYYHKVLVEARAARTEVLRGKDVSVRRR--
NCU00310.1	DPTATIGQKMEAHFNMGRAYSLIGLGNLAGGFYRRVLEEAERVRNRNQEGEEESTAEKRS
FG05376.1 	PVQDSSQNKAEAYYNLGRLYQLLGINYLALEYYSRAKKVLQSHRG---------------
AN0407.1  	--GTPLVERQEMEFNFARVWHTLGLAHLAVEGYQRVLDLGKEIQAQFQTKQLLTPAHAAN
          	    .  :: *  :*..* :  :*:  **   * :.    .  :      .  :     .

MG06314.1 	--------------------------------------------EDLSLESAM~NIRTYC
NCU00310.1	-----------------------------------------RWEETLVVEAAY~NVRCMC
FG05376.1 	--------------------------------------------HADALGGLE0NNKALP
AN0407.1  	GAQADDNDADISMPDVDSNVNIDVNVDRATHSVEVKPQSPQPVIENFSREAAV~ALQQLY
          	.:.:....:. : .. .:. . . . . :: : . ...:..   .     .     :   

MG06314.1 	LSTGDLEGARAVTDEWLVLE
NCU00310.1	FLLGDVKGAKAVAERWLVLE
FG05376.1 	LLKGRIKL------------
AN0407.1  	ALNGDFQSAQAVTAAWLVI-
          	   * .: : : :
```
